# YOU CAN’T GET THERE FROM HERE! TRANSPORTATION ISSUES OF OLDER ADULTS AND PEOPLE WITH DISABILITIES

**DOI:** 10.1093/geroni/igz038.948

**Published:** 2019-11-08

**Authors:** Angela M Zell, Joan Ilardo

**Affiliations:** Michigan State University College of Human Medicine, East Lansing, Michigan, United States

## Abstract

Older adults and individuals with disabilities face transportation challenges on a daily basis that differ from the general population. Transportation is critical to all aspects of quality of life yet presents significant challenges. Lack of appropriate vehicles, reliable public transportation, and the high-cost of vehicle ownership lead to missed healthcare appointments, lack of access to proper nutrition, and social isolation. The purpose of the study was to provide information for the Commission on Services to the Aging. It was necessitated by the lack of existing data from the Department of Transportation because Michigan’s transportation systems are locally controlled. An online questionnaire was emailed to public and private organizations serving older adults and people with disabilities to determine transportation services currently available in their geographic areas and innovative solutions employed to address barriers. The questionnaire was adapted from the National Center of Senior Transportation in 2009 that produced the report “Transportation: The Silent Need”. The study analyzed data on current transportation services and innovative solutions piloted in Michigan. Respondents represented every Michigan county and included area agencies on aging, senior centers, councils on aging, healthcare agencies, transportation providers, community action agencies, and job training programs. Information includes: services provided by the agency; barriers to accessing transportation; access to transportation services information; transport services previously used, currently available, and being planned. Many of the 95 respondents commented on persistent lack of funding for viable, reliable transportation options and jurisdictional issues. Most pilots used fixed routes, volunteer drivers, demand response and expanded schedules.

